# Effect of amplitude correlations on coherence in the local field potential

**DOI:** 10.1152/jn.00851.2013

**Published:** 2014-04-30

**Authors:** Ramanujan Srinath, Supratim Ray

**Affiliations:** Centre for Neuroscience, Indian Institute of Science, Bangalore, India

**Keywords:** amplitude correlations, coherence, local field potential

## Abstract

Neural activity across the brain shows both spatial and temporal correlations at multiple scales, and understanding these correlations is a key step toward understanding cortical processing. Correlation in the local field potential (LFP) recorded from two brain areas is often characterized by computing the coherence, which is generally taken to reflect the degree of phase consistency across trials between two sites. Coherence, however, depends on two factors—phase consistency as well as amplitude covariation across trials—but the spatial structure of amplitude correlations across sites and its contribution to coherence are not well characterized. We recorded LFP from an array of microelectrodes chronically implanted in the primary visual cortex of monkeys and studied correlations in amplitude across electrodes as a function of interelectrode distance. We found that amplitude correlations showed a similar trend as coherence as a function of frequency and interelectrode distance. Importantly, even when phases were completely randomized between two electrodes, amplitude correlations introduced significant coherence. To quantify the contributions of phase consistency and amplitude correlations to coherence, we simulated pairs of sinusoids with varying phase consistency and amplitude correlations. These simulations confirmed that amplitude correlations can significantly bias coherence measurements, resulting in either over- or underestimation of true phase coherence. Our results highlight the importance of accounting for the correlations in amplitude while using coherence to study phase relationships across sites and frequencies.

signals recorded from the brain often exhibit oscillatory patterns at different frequencies (Buzsáki 2006; Buzsáki and Draguhn 2004), which have been hypothesized to play a functional role in feature binding (Singer 1999) or to form communication channels across distinct brain areas (Fries 2005; Womelsdorf et al. 2007). A popular measure for studying such temporal correlations across brain areas is coherency, a complex spectral measure that, in brief, is the cross-spectrum (the Fourier transform of the cross-covariance function) between two signals normalized by the product of the auto-spectra (Jarvis and Mitra 2001; Mitra and Pesaran 1999). The modulus of coherency, called coherence, is generally considered to be a measure of the consistency in phase differences across trials between two signals (see materials and methods for mathematical details). Coherence, however, suffers from several biases. First, it depends on the number of samples (Maris et al. 2007; Vinck et al. 2010, 2012). Furthermore, coherence does not depend on phase consistency alone; it also depends on amplitude covariations (Lachaux et al. 1999). Because of common input, spiking activity of two brain regions is correlated, both spatially and functionally (Kohn and Smith 2005; Smith and Kohn 2008), so it is expected that the amplitude of the brain signals recorded from two areas should also show spatial and functional correlations, which will make it difficult to assess phase consistency with coherence. Although several studies have acknowledged this problem (see discussion), the magnitude of amplitude covariations and its effect on coherence have not been well characterized.

We address these issues by recording the local field potential (LFP) from chronically implanted 10 × 10 microelectrode arrays in monkeys. Microelectrodes were spaced 400 μm apart, allowing us to compute coherence between pairs of electrodes spaced at various distances from each other ranging from 400 μm to ∼4 mm. We evaluated the effect of amplitude correlations on field-field coherence, during spontaneous activity as well as during visual stimulation that generated a salient gamma rhythm. Furthermore, we simulated pairs of sinusoids with varying levels of phase consistency and amplitude correlations to study the dependence of coherence on these factors.

## MATERIALS AND METHODS

### 

#### Ethics statement.

The animal protocols used in this study were approved by the Institutional Animal Care and Use Committee of Harvard Medical School.

#### Recordings.

Recordings were made from two male rhesus monkeys (*Macaca mulatta*). Before training, a scleral search coil and a head post were implanted. After monkeys learned the behavioral task (∼4 mo), we first implanted the microelectrode array (Blackrock Microsystems, 96 active electrodes) in V1 of the right cerebral hemisphere (about 15 mm anterior from the occipital ridge and 15 mm lateral from the midline). The microelectrodes were 1 mm long and 400 μm apart, with an active electrode region made of platinum, a tip radius of 3–5 μm, and mean impedance of ∼0.9 MΩ (range: 0.2–1.8 MΩ) at 1 kHz. The entire length of the microelectrodes was inserted into cortex; we expect them to be in lower layer 2/3 or 4. Histology has not been performed. Signals were extracted with commercial hardware and software (Blackrock Microsystems), referenced to a wire placed on the dura near the electrode grid. Raw data were filtered between 0.3 Hz (Butterworth filter, 1st order, analog) and 500 Hz (Butterworth, 4th order, digital) and digitized at 2 kHz (16-bit resolution). The receptive fields of the neurons recorded from the microelectrodes were in the lower left quadrant of the visual field at an eccentricity of ∼3–5°. Results with the same monkeys and arrays have been reported elsewhere (Ray and Maunsell 2010, 2011).

#### Behavioral task.

The orientation-change detection task used here has been detailed in previous work (Ray and Maunsell 2011). Briefly, the monkey was required to hold its gaze within 1° of a small central dot (0.05–0.10° diameter) located at the center of a CRT video display (100-Hz refresh rate, 1,280 × 768 pixels, gamma corrected) while two achromatic, odd-symmetric stimuli were synchronously flashed for 400 ms with an interstimulus period of 600 ms, with one stimulus centered on the receptive field of one of the recorded sites (new location for each session) and the second stimulus located at an equal eccentricity on the opposite side of the fixation point. The monkey was cued to attend to the stimulus outside the receptive field for all the trials, which was a Gabor stimulus with an SD of 0.5°. At an unsignaled time (drawn from an exponential distribution), the orientation of the stimulus at the cued location changed by 90° and the monkey was rewarded with a drop of juice for making a saccade to the location of the changed stimulus within 500 ms of the orientation change. In the unattended hemifield, the stimuli were static gratings, with a spatial frequency of 4 cycles/degree (cpd), ∼100% contrast, located at the center of the receptive field of one of the sites (different recording site each session). They were presented at six possible orientations, 0°, 30°, 60°, 90°, 120°, and 150°, chosen pseudorandomly. The size of the grating stimuli could take six possible values, but we only considered the largest grating (radius of 2.4°) such that all the sites in the array were well stimulated. On average, each orientation was repeated 31 times (range 22–48) for *monkey 1* and 22 times (range 16–32) for *monkey 2*. We used the same electrode selection criteria as in previous papers (Ray and Maunsell 2010, 2011). Only electrodes with reliable estimates of the receptive field center were used (SD < 0.1° across days, mapped by flashing small Gabor stimuli on a rectangular grid that spanned the receptive fields of all the electrodes), yielding 27 and 66 electrodes from *monkeys 1* and *2*, respectively (1 region of the array did not yield usable signals in *monkey 1*). *Monkeys 1* and *2* performed the task in 10 and 24 sessions, respectively. We only considered sessions in which the receptive fields of all the selected sites (27 and 66 for the 2 monkeys) were within 1.8° of the stimulus center such that the stimulus (a grating of radius 2.4°) completely encompassed the receptive fields of all the sites. This was true in 10 and 17 sessions for *monkeys 1* and *2*, respectively. All analyses were done for an individual session separately, and the results were averaged across sessions.

#### Data analysis.

**Fig. 1.**
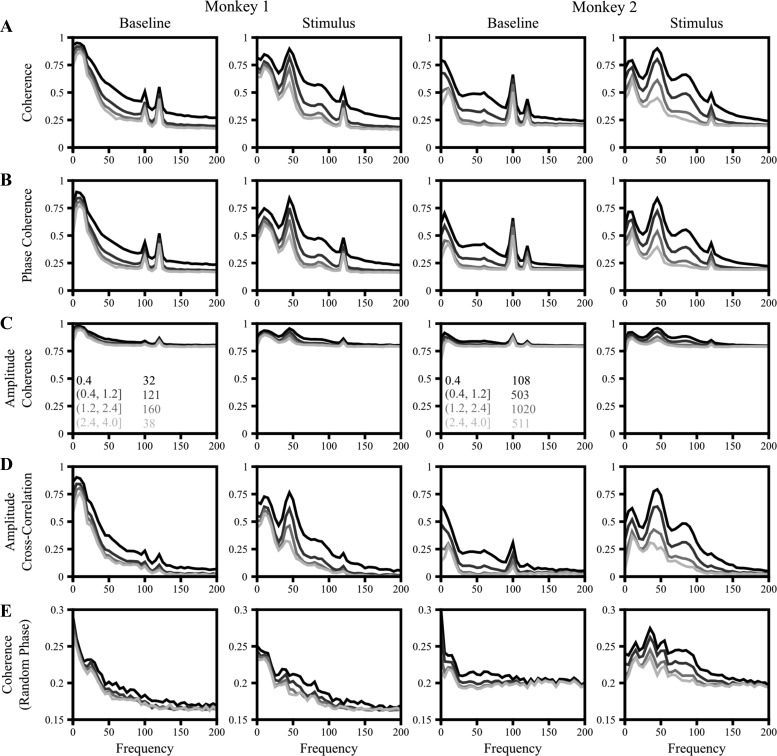
Coherence measures. *A*: coherence spectrum (*Eq. 1*) for electrode pairs with varying interelectrode distance, shown in shades of gray. Interelectrode distance ranges (in mm) and the number of pairs averaged for each range are given in *insets* in *C*. Baseline corresponds to the 300 to 100 ms interval before stimulus onset, while Stimulus epoch corresponds to the 200 to 400 ms interval after stimulus. *B*: phase coherence or phase locking value, computed after setting all amplitudes to 1 (*Eq. 2*). *C*: amplitude coherence, computed by setting all phase differences to 0 (*Eq. 3*). *D*: amplitude cross-correlation, which is the same as amplitude coherence but after first subtracting the mean amplitude across trials (*Eq. 4*). *E*: amplitude coherence after randomizing the phases (*Eq. 5*). Note the change in scale in this plot compared with *A–D*.

Coherency was computed with the multitaper method (Thomson 1982) implemented in Chronux 2.0 (Bokil et al. 2010), an open-source, data analysis toolbox available at http://chronux.org. Briefly, the multitaper method reduces the variance of spectral estimates by premultiplying the data with several orthogonal tapers known as Slepian functions (Jarvis and Mitra 2001; Mitra and Pesaran 1999). We used a single taper to maximize the frequency resolution. For each session, data from six different orientations were analyzed separately and the results were averaged across orientations to minimize variability arising because of dissimilar orientation preference of neurons. Analyses done after pooling all orientations yielded similar results. The baseline analysis was performed on the epoch 300 ms to 100 ms before the presentation of the stimulus. The stimulus analysis was performed on the epoch 200 ms to 400 ms after the presentation of the stimulus. The 100-Hz monitor refresh rate and 60-Hz line noise and their harmonics are referred to as “noise” frequencies. All results are shown between 0 and 200 Hz because the coherence reached a steady state beyond 200 Hz (Fig. 1).

#### Coherence measures.

If only a single taper is used and the Fourier transforms of two tapered signals are denoted in polar coordinates as *A*_*k*_(*f*)*e*^*j*ϕ_*k*_(*f*)^ and *B*_*k*_(*f*)*e*^*j*θ_*k*_(*f*)^ (where *f* denotes the frequency and *k* = 1, 2, . . . , *N* denotes the trial number), the coherence at frequency *f* is defined as

(1) $$C(f)=\left|\frac{\underset{k}{\sum }A_{k}(f)B_{k}(f)e^{j\left(\phi_{k}(f)-\theta_{k}(f)\right)}}{\sqrt{\underset{k}{\sum }A_{k}{\left(f\right)}^{2}}\sqrt{\underset{k}{\sum }B_{k}{\left(f\right)}^{2}}}\right|$$

Phase coherence, also called the phase locking value (Lachaux et al. 1999), is defined by setting amplitudes to 1.

(2) $$C_{\text{phase}}(f)=\frac{1}{N}\left|\underset{k}{\sum }e^{j\left(\phi_{k}(f)-\theta_{k}(f)\right)}\right|$$

Amplitude coherence is defined by setting phase difference to 0.

(3) $$C_{\text{amp}}(f)=\left|\frac{\sum_{k}A_{k}(f)B_{k}(f)}{\sqrt{\sum_{k}A_{k}{\left(f\right)}^{2}}\sqrt{\sum_{k}B_{k}{\left(f\right)}^{2}}}\right|$$

Cross-correlation between amplitudes is computed with *Eq. 3* but after subtracting the mean amplitude across trials.

(4) $$C_{\text{amp,meanDev}}(f)=\left|\frac{\sum_{k}\left(A_{k}(f)-A_{\text{mean}}(f))(B_{k}(f)-B_{\text{mean}}(f)\right)}{\sqrt{\underset{k}{\Sigma }{\left(A_{k}(f)-A_{\text{mean}}(f)\right)}^{2}}\sqrt{\sum_{k}{\left(B_{k}(f)-B_{\text{mean}}(f)\right)}^{2}}}\right|$$

where *A*_mean_(*f*) and *B*_mean_(*f*) are the mean amplitudes across *N* trials.

Finally, coherence with random phase is computed by

(5) $$C_{\text{amp,randomPhase}}(f)=\left|\frac{\sum_{k}A_{k}(f)B_{k}(f)e^{j\Phi_{\text{rand}}}(f)}{\sqrt{\sum_{k}A_{k}{\left(f\right)}^{2}}\sqrt{\underset{k}{\Sigma }B_{k}{\left(f\right)}^{2}}}\right|$$

where Φ_rand_ is a value drawn from a uniform distribution between [−π, π].

#### Pairwise phase consistency.

Pairwise phase consistency (PPC) is an unbiased estimator of the square of the true phase consistency and is calculated as follows (Vinck et al. 2010):

(6) $$\text{PPC}(f)=\frac{2}{N(N-1)}\sum_{j=1}^{N-1}\sum_{k=j+1}^{N}\cos\left(\theta_{j}(f)-\phi_{k}(f)\right)$$

#### Current source density.

Spatial current source density (CSD) is computed by using the following differential equation:

$$\sigma \left(\frac{\partial^{2}\phi }{\partial x^{2}}+\frac{\partial^{2}\phi }{\partial y^{2}}+\frac{\partial^{2}\phi }{\partial z^{2}}\right)=-\text{CSD(}x,y\text{,}z\text{)}$$

where ϕ is the electric potential at a point (*x*,*y*,*z*), σ is the conductivity of the extracellular medium, and CSD is the current source density at the same point (Einevoll et al. 2013). For laminar recordings in which electrodes are positioned along the *z*-axis, CSD can be approximated by a discrete double spatial derivative:

$$\text{CSD}(z_{j})=-\sigma \left(\frac{\phi \left(z_{j}+\delta \right)-2\phi (z_{j})+\phi \left(z_{j}-\delta \right)}{\delta^{2}}\right)$$

where δ is the interelectrode distance. For our two-dimensional microelectrode array, we took all electrodes that had four neighbors at 400 μm (17 and 29 electrodes for the 2 monkeys) and computed CSD as

(7) $$\text{CSD}\left(x,y\right)=V(x,y)-\frac{V\left(x-d,y\right)+V\left(x+d,y\right)+V\left(x,y+d\right)+V\left(x,y-d\right)}{4}$$

This is simply an extension of the previous equation in two dimensions. We did not multiply by σ or divide by δ^2^ because these terms cancel out while computing the coherence.

## RESULTS

All analyses were done separately for each monkey and for baseline (300 ms to 100 ms before stimulus presentation) and stimulus (200 ms to 400 ms after stimulus presentation) epochs.

### 

#### Amplitude correlation and its effect on coherence.

First, we studied correlations in the phases and amplitudes of microelectrodes separated by varying distances. Figure 1*A* represents the mean coherence (defined in *Eq. 1*) between the LFPs recorded from two electrodes separated by varying distances (interelectrode distance ranges and number of electrodes in each range are shown in Fig. 1*C*). We observed that for all interelectrode distance ranges, the coherence fell sharply between 0 Hz and 70 Hz. After 100 Hz, it was reasonably constant at a value around 0.25 (2 prominent peaks at 100 and 120 Hz are due to the monitor refresh rate and the second harmonic of noise). A significant bump in the gamma range (∼50 Hz) was also observed in the coherence plots for the stimulus epoch. In addition, there was a second gamma peak around ∼90 Hz, which was more prominent for *monkey 2*. These results are consistent with previous studies (Jia et al. 2011).

Although generally taken to be a measure of phase consistency, coherence depends on both phase and amplitude correlations. We therefore analyzed the components of the coherence spectrum starting with the phase consistency. The phase coherence (*Eq. 2*), also called the phase locking value (Lachaux et al. 1999), is a measure of the consistency of phase differences between two electrodes across trials, calculated by forcing the amplitudes of the signals at each frequency to 1 so as to remove any effects of covariations in amplitudes on the coherency. We observed that the phase coherence (Fig. 1*B*) was similar to the coherence observed in Fig. 1*A*, although phase coherence values were slightly less than coherence, which suggests that amplitude covariations might be playing a role as well.

To test this, we calculated the amplitude coherence by forcing the phase difference of the LFP signals at each frequency to 0 (*Eq. 3*). As shown in Fig. 1*C*, we observed that amplitude coherence also followed a trend similar to that observed in the coherence spectrum: both fell rapidly with frequency to a settle at a constant value, both had significant bumps in the gamma range when a stimulus was presented, and both fell with higher interelectrode distance.

**Fig. A1.**
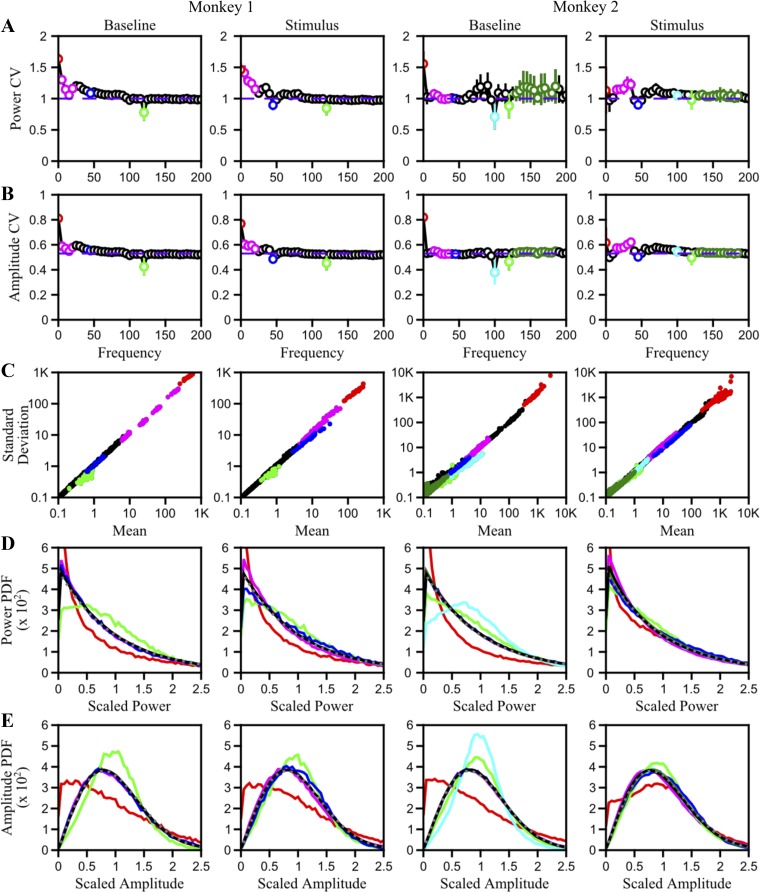
Amplitude distribution measures. *A*: coefficient of variation (CV; ratio of standard deviation and mean) for spectral power as a function of frequency. Black circles indicate CV across trials averaged for all electrodes at each frequency, and error bars indicate standard deviation in CV across electrodes. Frequencies at which CVs deviated from the theoretical value of 1 (violet line) are shown in different colors. (See *Extended Results* for more details.) *B*: CV for amplitudes as a function of frequency. Highlighted frequencies are same as those in *A*. Violet dashed horizontal line at ∼0.53 indicates the theoretical CV for Rayleigh distributions. *C*: standard deviation vs. mean scatterplot for spectral power. Each dot indicates the standard deviation and mean of power across trials for 1 electrode (27 and 66 dots per frequency for the 2 monkeys) using the same color scheme as in *A*. *D*: probability density function (PDF) of scaled spectral power at different frequencies as indicated in *A*. Gray dotted line denotes the distribution for the theoretical exponential distribution. *E*: same as *D*, for local field potential amplitude. Gray dotted line denotes the distribution for the theoretical Rayleigh distribution.

Although amplitude coherence showed a trend similar to that of phase coherence, the range was much smaller. This is because amplitudes are always positive and therefore their means are not 0. When a single taper is used as we did here, the multitaper estimate of the amplitude follows a Rayleigh distribution (see Fig. A1 in appendix). For two independent Rayleigh distributions, as per *Eq. 3*, the value of amplitude coherence is π/4 = ∼0.78 (derived in appendix), which is similar to the values beyond ∼100 Hz in Fig. 1*C*. This means that amplitude coherence can effectively vary only between ∼0.78 and 1. To address this limitation, we recomputed the amplitude coherence after first subtracting the mean amplitude (*Eq. 4*), as shown in Fig. 1*D*. After mean subtraction, this is simply the cross-correlation between amplitudes. The amplitude cross-correlation indeed showed a trend very similar to the phase coherence (Fig. 1*B*).

Finally, to study the amount by which coherence can change solely because of amplitude covariations, we recomputed the coherence after randomizing the phase differences (*Eq. 5*, Fig. 1*E*). Note that the scale on the *y*-axis is smaller in than other figures. Nonetheless, coherence changed by more than ∼0.1 going from low to high frequencies as the amplitude correlation decreased from ∼0.8 to 0 (most salient in the baseline condition for *monkey 1*). These results suggest that when a moderate number of trials are used, small changes in coherence across frequencies or experimental conditions could theoretically be due to changes in amplitude correlations alone.

#### Simulations.

**Fig. 2.**
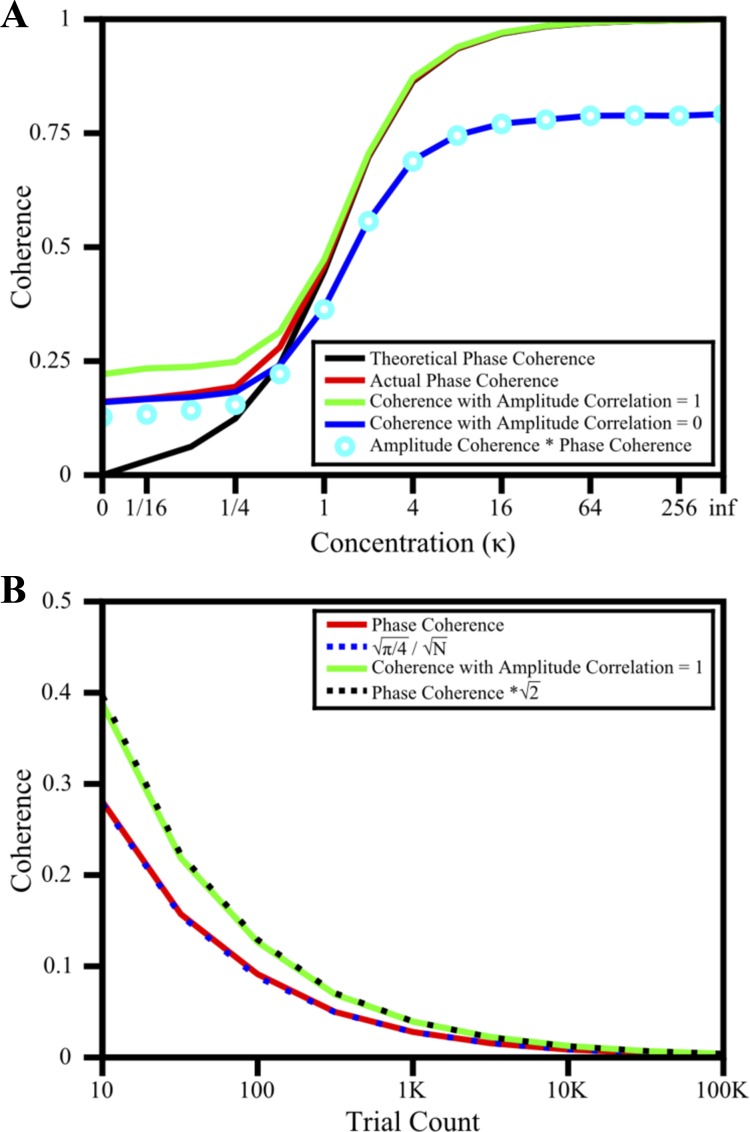
Simulations. *A*: coherence measures as a function of concentration parameter (κ) of the von Mises distribution varying from 0 (uniformly distributed phase differences) to infinity (all phase differences set to 0): theoretical phase coherence (black), actual phase coherence (*Eq. 2*; red), coherence [*Eq. 1*; amplitude correction (*A*_cc_) = 1; green], coherence (*Eq. 1*; *A*_cc_ = 0; blue), and $C_{\text{amp}}\;\times \;C_{\text{phase}}=\frac{\pi }{4}\;\times \;C_{\text{phase}}$ (cyan circles). *B*: phase coherence (*Eq. 2*; red) and coherence (*Eq. 1*; *A*_cc_ = 1; green) as a function of trial count. Mathematical approximations (see appendix) of phase coherence and coherence (*A*_cc_ = 1) shown by blue and black dotted lines.

Although Fig. 1*E* clearly shows the effect of amplitude correlations on coherence, the magnitude of this effect and the underlying causes are difficult to determine because both phase and amplitude correlations (Fig. 1, *B* and *D*) have a similar profile versus frequency. We therefore performed a series of simulations to study how amplitude correlations affect coherence at different levels of phase consistency. We first studied the distributions of amplitude and power across trials and found that they followed Rayleigh and exponential distributions respectively (see Fig. A1 and *Extended Results* in appendix). This stereotypical distribution is enforced by the spectral estimator and has no neurophysiological basis. For these simulations, we fixed the number of trials to 25, which is the average in our data set and is a typical number in many neurophysiological studies. Phase differences were derived from a von Mises distribution with mean zero and varying concentration parameter (κ). For this distribution of phase differences, the true phase coherence is given by $C_{\text{phase}}=\frac{I_{1}(K)}{I_{0}(K)}$, where *I*_0_ and *I*_1_ are modified Bessel functions of the first kind (Fisher 1993), as shown by the black trace in Fig. 2*A*. The phase coherence of the simulated trials showed a positive bias at low values. As described previously (Vinck et al. 2010, 2012), this bias arises because the numerator of coherence is the mean of phase vectors multiplied by the product of amplitudes, and for a small number of trials these vectors do not average out even when pointing at random directions.

We studied the effect of amplitude correlations on coherence by computing the coherence under two cases—first when the amplitudes were derived from two independent Rayleigh distributions (blue trace; amplitude correlation is 0), and when both amplitudes were equal to each other (green trace; amplitude correlation is 1). In both cases, we assumed that phases and amplitudes were independent of each other. In the first case, coherence was smaller than phase coherence at high values of κ. This can be explained as follows. If phases are independent of the amplitudes, the numerator of *Eq. 1* can be simplified as the product of expected values of the amplitude and phase terms i.e., *C* = *C*_amp_·*C*_phase_ (as derived in appendix). When amplitudes are derived from independent Rayleigh distributions, *C*_amp_ = π/4 ∼0.78, such that *C* = (π/4)·*C*_phase_. Indeed, (π/4)·*C*_phase_ was very close to the coherence values when phase coherence was high. At low phase coherence values, this relationship broke down and both coherence and phase coherence converged to the same value (we show in appendix that the bias for coherence and phase coherence is the same when amplitudes are independent), which only depended on the number of trials.

When both amplitudes are equal to each other, *C*_amp_ = 1, the coherence is expected to be equal to the phase coherence. This was indeed the case for large values of phase coherence, but for low values coherence converged to a larger value. This unexpected result can be explained as follows. When the amplitude for the first electrode is derived from a Rayleigh distribution and the second amplitude is always equal to the first, the product of amplitudes is simply the square of a Rayleigh distribution, which is an exponential distribution. The exponential distribution has a mode of 0, such that a large proportion of trials have very small values. When the phase vectors in the numerator of coherence are multiplied with values derived from an exponential distribution, a large fraction of terms drop out, effectively reducing the number of trials and therefore increasing the bias (we show in appendix that the bias increases by a factor of $\sqrt{2}$). This explains why once phases are randomized (as in Fig. 1*E*) coherence depends on the value of amplitude correlation and decreases as amplitude correlation decreases from ∼0.8 to ∼0.

In summary, there are two distinct processes that lead to deviation between coherence and phase coherence. First, if amplitudes of the two electrodes are independent, coherence is less than phase coherence and is approximately equal to (π/4)·*C*_phase_. This effect is more prominently observed at high phase coherence values and does not depend on the number of trials used to compute these measures. Second, when amplitudes are perfectly correlated and derived from a Rayleigh distribution, coherence has a larger bias than phase coherence, which is more prominently observed at low phase coherence values. This effect is due to finite sample size, and therefore should decrease with increasing number of trials.

We tested this prediction by computing, for randomized phases (κ = 0), the phase coherence and coherence with amplitude correlation set to 1 for varying number of trials (Fig. 2*B*). As expected, the bias decreased with an increase in the number of trials. In particular, *C*_phase_ was well described by $C_{\text{phase}}=\frac{\sqrt{\pi /4}}{\sqrt{N}}$ and $C_{\text{phase}}=\sqrt{2}\;\times \;C_{\text{phase}}$. These relationships are derived mathematically in the appendix.

On average we had 31 and 22 trials per orientation for the two monkeys, a typical number used in neurophysiological studies. We also calculated the coherence with randomized phases (as in Fig. 1*E*) after pooling all the trials from six orientations (for the baseline condition before the stimulus appears, it makes no difference), for which the average number of trials were 185 and 135 for the two monkeys. Consistent with the simulations, the curves in Fig. 1*E* were indeed scaled by $\frac{∼1}{\sqrt{6}}$, such that the maximum difference in coherence between low and high frequencies was now ∼0.05 (data not shown).

**Fig. 3.**
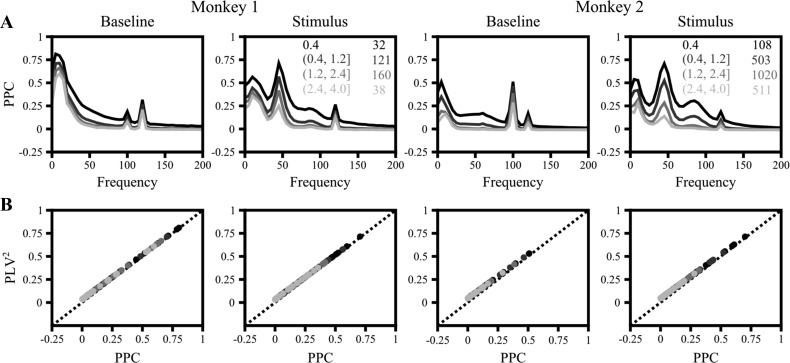
Pairwise phase consistency (PPC). *A*: PPC (*Eq. 6*) between pairs of electrodes with varying interelectrode distance, shown in shades of gray (as in Fig. 1). *B*: scatterplot of squared phase coherence (PLV^2^) and PPC. Each dot represents PLV^2^-PPC relationship at a particular frequency. Similarly colored dots belong to the same interelectrode distance range. Interelectrode distance ranges (in mm) and the number of pairs averaged for each range are given in *insets* in *A*.

Although the use of phase coherence instead of coherence eliminates the potential bias introduced by amplitude correlations, it does not eliminate the positive bias due to a small number of trials. To study the bias introduced by finite sample size in our data set in more detail, we compared phase coherence to PPC (*Eq. 6*), which gives an unbiased estimate of the square of the true phase coherence (Vinck et al. 2010). Figure 3*A* shows PPC as a function of frequency and distance (as in Fig. 1). PPC values converged to 0 at high frequencies, suggesting that phases indeed became random at these frequencies. To directly compare PPC with phase coherence, we plotted PPC versus the square of phase coherence (Fig. 3*B*). We observed that these two measures were very similar except when PPC was close to 0, which is due to the positive bias of phase coherence. Note that the bias appears to be much smaller here, but this is simply the effect of squaring. For example, in Fig. 1*B*, the minimum phase coherence for *monkey 1* is ∼0.16 (close to the theoretical value of 0.886/√31), which, after squaring, is only ∼0.03.

**Fig. 4.**
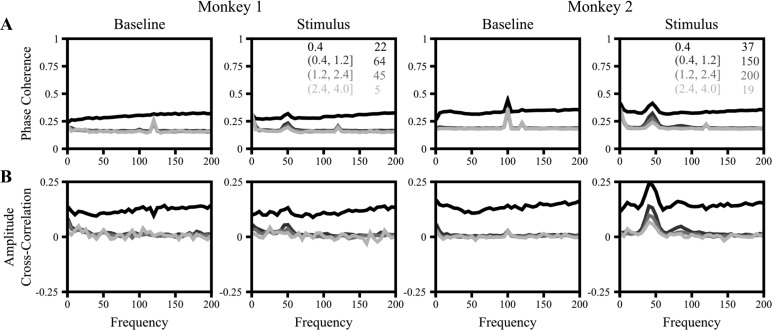
Current source density (*Eq. 7*). Phase coherence (*A*) and amplitude cross-correlation (*B*) as computed with current source densities, plotted as a function of frequency with varying interelectrode distances (in gray). Note the change in scale in *B* and the change in number of pairs of electrodes averaged for each range compared with Figs. 1 and 3.

Finally, we explored the potential reasons why amplitude and phase correlations showed similar trends in our data set. The simplest explanation is based on volume conduction—since the interelectrode distance is small, nearby electrodes pick up the activity of the same source, thus leading to trivial correlations in both amplitudes and phases that would decrease with increasing interelectrode distance. To test for this possibility, we first computed the CSD as a double spatial derivative of potential (*Eq. 7*) and performed the same analysis as Fig. 1 on CSDs instead of potentials. Figure 4, *A* and *B*, show the phase coherence and amplitude correlation plots (analogous to Fig. 1, *B* and *D*) for CSDs. Both these curves were almost flat at all frequencies, with values close to 0 except when the interelectrode distance was 400 μm. However, this is simply because of the way CSDs were computed: because we subtracted the four neighboring electrodes to compute the CSDs, two CSDs separated by 400 μm would have a common component (if the initial voltages were *V*_1_ and *V*_2_, we would have *V*_1_ − *V*_2_/4 term in *CSD1* and *V*_2_ − *V*_1_/4 in *CSD2*), leading to trivial correlations. These results confirm the role of volume conduction in the observed correlations in amplitudes and phases as shown above. Our emphasis in this report is only to show the effect of amplitude covariations on coherence, irrespective of the origins of these covariations. Note that CSD coherence is 0 everywhere except at gamma frequencies in the stimulus period, which suggests local origins of gamma sources. This suggests that there are multiple local sources of gamma that are coordinated globally such that there is nonzero coherence between the local sources. This is in line with a previous study that showed that gamma recorded from microelectrodes separated by as little as 400 μm oscillated at significantly different frequencies when a Gabor stimulus (whose local contrast varied in space) was presented, suggesting multiple sources with very local origin, but all these local sources oscillated in phase with high coherence when a grating was presented (suggesting global coordination when contrast was uniform; Ray and Maunsell 2010).

## DISCUSSION

We recorded LFP data from a 10 × 10 grid of microelectrodes implanted in V1 and studied covariations in amplitude across trials between pairs of electrodes separated by varying distances (between 0.4 and ∼4 mm) and the effect of this covariation on coherence, a popular measure for quantifying phase consistency. Amplitude covariations followed a specific profile with respect to frequency, and this trend was similar to phase coherence. These amplitude covariations had a significant effect on coherence, as shown by a finite coherence when phases of the pairs of electrodes were randomized across trials. The effect of amplitude correlations on coherence was further studied with simulations. First, we confirmed that power and amplitude followed exponential and Rayleigh distributions at most frequencies, which is due to the properties of the spectral estimator (discussed in more detail below). Simulating amplitudes using a Rayleigh distribution and phase differences from a von Mises distribution with different values of the concentration parameter, we found that coherence was greater than phase coherence when amplitude correlation was high and phase coherence was low, but the opposite effect was observed when phase coherence was high but amplitudes were uncorrelated. In particular, when the true phase coherence was 0 (phases followed a uniform distribution), coherence was equal to $\frac{\sqrt{\pi /4}}{\sqrt{N}}$ when amplitudes were uncorrelated but increased by a factor of $\sqrt{2}$ when amplitudes were perfectly correlated. We analytically derived these biases and their dependence on the number of trials. We characterized the dependence of phase coherence on the number of trials by comparing it to an unbiased metric called pairwise phase consistency (PPC) and showed that PPC was close to the square of phase coherence except when PPC was close to 0. Finally, using current source density (CSD) analysis, we showed that the similar profiles of phase coherence and amplitude correlations in our data set could be due to volume conduction effects.

### 

#### Factors that influence coherence.

The bias in coherence due to amplitude covariations has been acknowledged in several reports and is usually addressed by using the phase coherence (Lachaux et al. 1999; Mormann et al. 2000; Tallon-Baudry et al. 2004; Uhlhaas et al. 2006). However, both coherence and phase coherence are biased by sample size, which has recently been addressed by using PPC, which provides an unbiased estimator of the square of the phase coherence (Vinck et al. 2010) and revealed a true phase coherence of 0 at high frequencies in our data set. Coherence may also be influenced by volume conduction, which has been addressed by taking the phase lag index (PLI), which is a measure of the asymmetry of the distribution of phase differences between two signals (Aydore et al. 2013; Stam et al. 2007). In our data set, because we recorded from a grid of microelectrodes, we were able to directly compute the CSD, which removes volume conduction effects.

Note that we have only considered field-field coherence in this report, not spike-field coherence that is more popular in studies involving LFPs where spiking data are available (Fries et al. 2008; Gregoriou et al. 2009; Pesaran et al. 2008). If one of the two signals is a spike train, the overall firing rate (as well as the timing of the spikes) should influence the amplitude of this signal. Recent studies have shown that the spike-field coherence is indeed biased by the overall rate of spiking (Grasse and Moxon 2010; Lepage et al. 2011; Vinck et al. 2012). Note that the bias due to amplitude covariations is not eliminated by simply *z*-scoring the data (Pesaran et al. 2008; Schoffelen et al. 2005), a popular technique used to compare coherence values across different experimental conditions (Maris et al. 2007).

Another factor that can influence coherence is the signal-to-noise ratio (SNR). Any brain signal has some noise component—either of neurophysiological origin or related to instrumentation and data acquisition. Fourier coefficients of the LFP signal that are used for the computation of coherence will have a greater contribution from noise when SNR is low, and because the noise component is associated with low phase consistency, the overall coherence will be low. For example, if we assume that signal power decreases with increasing frequency but the noise is white (which has equal power at all frequencies), SNR and coherence will both decrease with frequency.

Unfortunately, testing the contribution of SNR on coherence through simulations requires the knowledge of “signal” and “noise” components of the LFP, which is not straightforward to estimate. Slope of the power spectral density, which may provide clues about the underlying sources and noise, is typically ∼2 (ranges between 1 and 3) at frequency ranges below 100 Hz, both in EEG and ECoG (Freeman et al. 2000, 2006; He et al. 2010; Miller et al. 2009; Pritchard 1992) as well as LFP (Bédard et al. 2006; Petermann et al. 2009), which could be due to shot (Brownian) noise arising from an exponential relaxation process of synaptic currents (such as slow dendro-synaptic decay) driven by random (Poisson) spiking (Bédard et al. 2006; Miller et al. 2009; Milstein et al. 2009) or correlated firing during alternating UP and DOWN states (Milstein et al. 2009).

#### Estimation of power spectrum.

The effect of amplitude correlations on coherence is critically dependent on the distribution of amplitude across trials, as shown in the appendix. The power spectral density is computed by simply taking the square of the modulus of the Fourier transform of the signal that has been premultiplied by the taper. This is a type of direct spectral estimator, whose properties have been studied extensively (see Jarvis and Mitra 2001 for a review). Unfortunately, such estimators have large variability, essentially because we are trying to estimate the true spectrum at an infinite number of points from a finite length of the signal. These power estimates are distributed as *S*(*f*)·χ_2_^2^/2 for asymptotic sample sizes (i.e., when the signal length approaches infinity), where *S*(*f*) is the true spectrum (Brillinger 2012; Jarvis and Mitra 2001). This is equivalent to an exponential distribution (as shown in Fig. A1). The square root of the exponential distribution yields a Rayleigh distribution with a CV of ∼0.53. Thus the measured variance can be attributed to the behavior of the estimator rather than to a physiological source.

Because direct spectral estimators have high variability, the estimator-induced variance is likely to mask any physiological variability. This is a serious issue when single-trial estimates are used for predicting behavior [for example, the reaction time in a change detection task (Womelsdorf et al. 2007)]. This issue can be partially addressed by smoothing the direct spectral estimator with a smoothing kernel (called a lag window estimator) or by with multiple orthogonal tapers (Jarvis and Mitra 2001). However, because in such techniques we take a weighted average of the power across a larger frequency range (determined by the width of the smoothing kernel in case of a lag window estimator and the time-frequency bandwidth product in case of the multitaper estimator), the frequency resolution is poorer.

In conclusion, this study shows significant coupling in the amplitudes across sites and frequencies and suggests cautious usage of coherence as a measure of phase consistency because of the contribution of amplitude covariations. This is especially important because in neurophysiological signals changes in coherence across stimulus or behavioral conditions are typically low. For example, even when a salient gamma rhythm is induced by presenting a large high-contrast stimulus, coherence rarely changes from baseline levels by more than ∼0.2 (Jia et al. 2013; Ray and Maunsell 2010; Roberts et al. 2013). Changes in coherence in cognitive tasks, such as during a shift in attention, are typically less than ∼0.1 (Bosman et al. 2012). Note that these small values could be due to the presence of noise in the recording system or the short-lived nature of basic neuronal events (action potentials and postsynaptic potentials), which have broadband spectral signature; they do not necessarily imply that physiological gamma coupling is weak or absent. Nonetheless, while studying the effect of behavior such as attention on neural synchronization, it is essential to rule out the confounding effects of amplitude covariations because attention can also change the correlation in spiking across sites (Cohen and Maunsell 2009; Mitchell et al. 2009), which could potentially influence the magnitude of amplitude covariations across sites.

## GRANTS

This work was supported by Wellcome Trust/DBT India Alliance (Intermediate Fellowship to S. Ray) and startup funding provided by the Indian Institute of Science.

## DISCLOSURES

No conflicts of interest, financial or otherwise, are declared by the author(s).

## AUTHOR CONTRIBUTIONS

Author contributions: R.S. and S.R. analyzed data; R.S. and S.R. interpreted results of experiments; R.S. and S.R. prepared figures; R.S. and S.R. drafted manuscript; R.S. and S.R. edited and revised manuscript; S.R. conception and design of research; S.R. performed experiments; S.R. approved final version of manuscript.

## Appendix

### Amplitude Coherence Asymptote

The asymptotic value of ∼0.78 for the amplitude coherence (Fig. 1*C*) can be computed analytically based on the properties of the amplitude distribution. We denote the amplitudes of the two electrodes by random variables *A* and *B*. If the amplitude *A* ∼ *Rayleigh*, then $E(A)=\sigma_{a}\sqrt{\frac{\pi }{2}}$ and $\text{var}(A)=\left(\frac{4-\pi }{2}\right)\sigma_{a}^{2},$ where σ_*a*_ is the distribution parameter. Using *E*(*A*^2^) = var(*A*) + (*E*(*A*))^2^, we get *E*(*A*^2^) = 2σ_*a*_^2^. Hence $\frac{E(A)}{\sqrt{E{\left(A\right)}^{2}}}=\sqrt{\frac{\pi }{4}}$. Similarly, *E*(*B*^2^) = 2σ_*b*_^2^ and $\frac{E(B)}{\sqrt{E{\left(B\right)}^{2}}}=\sqrt{\frac{\pi }{4}}$.

If *A* and *B* are uncorrelated (which happens at high frequencies, see Fig. 1*D*), then *E*(*AB*) = *E*(*A*)*E*(*B*) and the amplitude coherence (*Eq. 3*) reduces to $\frac{E(A)}{\sqrt{E{\left(A\right)}^{2}}}\;\frac{E(B)}{\sqrt{E{\left(B\right)}^{2}}}=\frac{\pi }{4}\approx 0.78$.

### Relationship Between Coherence and Phase Coherence

If the product of amplitudes is independent of the phases, coherence (*Eq. 1*) is simply the product of phase coherence (*Eq. 2*) and amplitude coherence (*Eq. 3*). Specifically, if *X* = *AB* and *Y* = *e*^*j*ϕ^, where *A* and *B* are the amplitudes of the two electrodes and ϕ indicates the phase difference, then the numerator of *Eq. 1* is simply

N.E(XY)=N.E(X.Real(Y)+jX.Imag(Y))=N.[E(X)E(Real(Y))+jE(X)E(imag(Y))] (because of independence) =N.E(X).[E(Real(Y))+jE(imag(Y))]=N.E(X).E(Y).

Therefore,

$$\frac{N.E(XY)}{\sqrt{E(A^{2})E(B^{2})}}=\frac{N.E(X).E(Y)}{\sqrt{E(A^{2})E(B^{2})}}=E(Y)\frac{N.E(AB)}{\sqrt{E(A^{2})E(B^{2})}}=C_{\text{phase}}\cdot C_{\text{amp}}$$

since *E*(*Y*) is the phase coherence and *N.E*(*X*) divided by the denominator is the amplitude coherence.

When *A* and *B* are independent, *C*_amp_ = π/4 (as proved above); thus $C=\frac{\pi }{4}\cdot C_{\text{phase}}$.

### Dependence on Number of Trials

Central to the following proofs is the fact that the sum of squares of *k* independent standard normal distributions is a χ_*k*_^2^-distribution with *k* degrees of freedom, which, for *k* = 2, is an exponential distribution with rate parameter of λ = 1/2. Therefore, the square root of the sum follows a Rayleigh distribution with scale parameter of 1. This can be extended to show that given two independent random variables *X* and *Y* that are both normally distributed with zero mean and variance of σ^2^, the variable $Z=\sqrt{X^{2}+Y^{2}}$ is Rayleigh distributed with scale parameter of σ.

### Phase Coherence

From *Eq. 2*:

$$C_{\text{phase}}=\frac{1}{N}\left|\overset{N}{\underset{k=1}{\sum }}e^{j\theta }k\right|=\sqrt{{\left(\frac{\sum_{k=1}^{N}\cos(\theta_{k})}{N}\right)}^{2}+{\left(\frac{\sum_{k=1}^{N}\sin(\theta_{k})}{N}\right)}^{2}}$$

Here we consider $X=\frac{\sum_{k=1}^{N}\cos(\theta_{k})}{N}$ and $Y=\frac{\sum_{k=1}^{N}\sin(\theta_{k})}{N}$. When phases are derived from a uniform distribution, cos(θ_*k*_) and sin(θ_*k*_) are independent across trials, and therefore by central limit theorem both *X* and *Y* approach a normal distribution. Mean(*X*) = 0 and mean(*Y*) = 0. $\mathrm{var}(X)=\mathrm{var}(Y)=\frac{1}{2N}$. This is because var(*X*) = *E*(*X*^2^) because *E*(*X*) is 0, $E(\cos{\left(\theta_{k}\right)}^{2})=\frac{1}{2}$ (there are *N* such terms in the sum, and all cross terms have an expected value of 0).

As discussed above, *C*_phase_^2^ follows a χ_2_^2^ distribution such that

$$\frac{C_{\text{phase}}^{\text{2}}}{\text{1/2}N}=\frac{X^{2}}{\text{1/2}N}+\frac{Y^{2}}{\text{1/2}N}\sim \chi_{2}^{2}\sim Exp\left(\lambda =\frac{1}{2}\right)$$

Now, if *Z* ∼ χ_2_^2^ then $\sqrt{Z}$ follows a Rayleigh distribution with scale parameter of 1, for which the mean is $\sqrt{\frac{\pi }{2}}$ (Formally, $E(\sqrt{Z})=\int_{0}^{\infty }\sqrt{z}e^{-\frac{z}{2}}dz$ = $\sqrt{\frac{\pi }{2})}$

$$∴\;E(C_{\text{phase}})=\frac{1}{\sqrt{2N}}\sqrt{\frac{\pi }{2}}\approx \frac{0.886}{\sqrt{N}}$$

In general, if we can put coherence in the format above (in terms of *X* and *Y*), the expected value (bias) is simply the standard deviation of *X* multiplied by √π/2.

### Coherence

Without loss of generality, we assume that the amplitudes are Rayleigh distributed with a scale parameter of 1 (it can easily be shown that a different scale parameter changes the numerator and denominator of coherence by the same amount and therefore cancels out). Therefore *E*(*A*) = √π/2 and *E*(*A*^2^) = 1/λ = 2.

The denominator in *Eq. 1* is the square root of the sum of the square of amplitudes, i.e., $\sqrt{\sum_{k=1}^{N}a_{k}^{2}}$, and therefore is itself a random variable. However, we use the fact that a sum of *N* independent exponential distributions (each with parameter 1) has a gamma distribution with shape parameter *N* and rate parameter of 1, for which both mean and variance is *N*. Therefore, the standard deviation increases only as √*N*, and the standard error in the mean (= *stdDev*/*N*) decreases as 1/√*N*. We therefore approximate the denominator by its expected value, which is simply √2*N* for each amplitude term and 2*N* overall. We found in our simulations that coherence was indeed Rayleigh distributed, which validates this approximation made here.

The difference between the uncorrelated and equal amplitude cases can be summarized as follows. If *A* and *B* are uncorrelated, *E*(*A*^2^*B*^2^) = *E*(*A*^2^)*E*(*B*^2^) = 2 × 2 = 4. However, when *A* = *B*, *E*(*A*^2^*B*^2^) = *E*(*A*^4^) = 8, i.e., the numerator increases by a factor of 2 even though the denominator does not change. This causes an increase of √2 in the bias. Formal proofs follow.

### Case 1: Amplitudes are independent.

If $X=\sum_{k=1}^{N}a_{k}b_{k}\cos\left(\theta_{k}\right)/2N$ (the 2*N* term appears in the denominator as explained earlier), then *E*(*X*) = 0 and

$$E(X^{2})=\left(\frac{1}{4N^{2}}\right)E\left(\overset{N}{\underset{k=1}{\sum }}a_{k}^{2}b_{k}^{2}\cos^{2}\theta_{k}\right)\;\;\;=\left(\frac{1}{4N^{2}}\right)\cdot \overset{N}{\underset{k=1}{\sum }}E\left(a_{k}^{2}\right)E\left(b_{k}^{2}\right)E(\cos^{2}\theta_{k})=\left(\frac{1}{4N^{2}}\right)\left(N\cdot 2\cdot 2\cdot \left(\frac{1}{2}\right)\right)=\frac{1}{2N}$$

This is the same as the phase coherence case. Thus coherence and phase coherence have the same bias when amplitudes are independent (blue and red traces in Fig. 2*A* converge to the same value).

### Case 2: Amplitudes are the same.

$$X=\sum_{k=1}^{N}a_{k}^{2}\cos\left(\theta_{k}\right)/2N$$

, where *A* ∼ *Rayleigh* with parameter 1.

$$\text{Then}\;{\text{A}}^{\text{2}}/2\sim Exp\left(\lambda =1\right)\;\text{i.e.}\;{\text{A}}^{\text{2}}\sim 2\cdot Exp(\lambda =1).$$

$$\text{Now}\;\text{if}\;Z=A^{2}\;\text{then}\;E(Z^{2})=4\cdot E(Exp\left(\lambda =1\right))=8.$$

$$\text{Hence}\;E(X^{2})=\left(\frac{1}{4N^{2}}\right)\cdot N\cdot 8\cdot \left(\frac{1}{2}\right)=\left(\frac{1}{N}\right).$$

Thus the standard deviation (and hence the bias) increases by a factor of $\sqrt{2}$.

### Extended Results

We studied how power and amplitude are distributed across trials. Because both mean and standard deviation of power/amplitude are frequency dependent and follow a “1/f” form, we used the ratio of standard deviation and mean, called the coefficient of variation (CV), for analysis. Figure A1, *A* and *B*, shows the CV of power and amplitude, respectively, across trials. We observed that CV across trials for power and amplitude were tightly clustered around 1 and ∼0.53, respectively, for a wide range of frequencies. This tight clustering of CV does not have any neural basis. Instead, it is because of the properties of the spectral estimator, due to which power and amplitude follow exponential and Rayleigh distributions, respectively. Interestingly, CV across trials deviated from the theoretical values at certain frequencies, which was more evident for power than amplitude CVs. As shown in Fig. A1*A*, for *monkey 1*, power CVs were higher than 1 at 0 Hz and low frequencies (5–25 Hz; pronounced during stimulus epoch). For *monkey 2*, power CVs were higher than 1 at 0 Hz and high frequencies (>100 Hz) during the baseline epoch and low frequencies (15–40 Hz) for the stimulus epoch. In contrast, CVs were <1 at the second harmonic of noise (120 Hz) for both monkeys and at monitor refresh rate during the baseline epoch for *monkey 2* (100 Hz). Most importantly, the CV was <1 at the peak of gamma frequency (45 Hz) during the stimulus epoch for both monkeys, where a salient dip in CV was observed. Similar (though weaker) trends were observed for the amplitude CVs (Fig. A1*B*).

To investigate these deviations in more detail, we made a scatterplot of the mean vs. standard deviation of the power (Fig. A1*C*). From Fig. A1*A* it follows directly that the points in the scatterplot should fall on a line with a slope of 1, irrespective of magnitude, which was indeed the case: linearity was maintained over four orders of magnitude. We also observed that the slope of the scatterplot for “noise” (light green in *monkey 1*; cyan in the baseline epoch of *monkey 2*) and gamma frequencies (for stimulus epoch; more salient in *monkey 1*) was less than the slope of the scatter at other frequencies.

We also plotted a histogram of the power at different frequencies (normalized by the mean so that all distributions had a mean of 1), as shown in Fig. A1*D*. The gray dotted line shows the theoretical exponential distribution and lies on top of the black curve. At frequencies at which the CV was >1 in Fig. A1*A* (red curve at both epochs and magenta curve at stimulus epoch), we observed that the distribution had more values near 0 than an exponential (and consequently more very large values as well, because the means are the same for both distributions), which resembled a gamma function with a shape parameter < 1. We observed the opposite trend at frequencies at which the CV was <1 (“noise” frequencies and the gamma range during the stimulus epoch), where the distribution had fewer values near 0 (and consequently fewer very large values) and resembled a gamma function with a shape parameter > 1. Similar results were observed for the amplitude distributions (Fig. A1*E*), where the peak of the Rayleigh distribution shifted to the left for frequencies that had amplitude CV above ∼0.53 and shifted to the right (and also sharpened) for noise and gamma frequencies where the CV was below ∼0.53.

It is unclear why the CVs are lower from the theoretical values at noise frequencies or the peak of the gamma (45 Hz), because the distribution of the estimate does not depend on the signal itself. It could be due to the sinusoidal nature of these signals (Fourier transform is application only for square integrable signals, but a sinusoid has infinite energy). Frequencies at which the CVs were higher than the theoretical limit could be because of high physiological variability in the signal at those frequencies, although CVs were never much greater than the theoretical limit.
